# Supplementary material for: Complex 33-beam simulated galactic cosmic radiation exposure impacts cognitive function and prefrontal cortex neurotransmitter networks in male mice
Source: Nat Commun. 2023 Nov 27;14:7779. doi: 10.1038/s41467-023-42173-x (PMC10682413; doi:10.1038/s41467-023-42173-x)
Supplement: Supplementary file 2 — Supplementary Information [file 41467_2023_42173_MOESM2_ESM.pdf]

Supplementary Information:

Complex 33-beam simulated galactic cosmic radiation exposure impacts cognitive function and prefrontal cortex neurotransmitter networks in male mice

Rajeev I. Desai[1,2,3]*, Brian D. Kangas[1,2], Oanh T. Luc[1,2], Eleana Solakidou[3,4], Evan C. Smith[3], Monica H. Dawes[1,2], Xiaoyu Ma[3], Alexandros Makriyannis[3], Subhamoy Chatterjee[5], Maher A. Dayeh[6,7], Andrés Muñoz-Jaramillo[5], Mihir I. Desai[6,7], Charles L. Limoli[8]

[1]*Department of Psychiatry, Harvard Medical School, Boston, MA 02115, USA (RID, BDK, OTL, MHD)*

[2]*Behavioral Biology Program, McLean Hospital, Belmont, MA 02478, USA (RID, BKD, OTL, MHD)*

[3]*Center for Drug Discovery, Department of Pharmaceutical Sciences, Northeastern University, Boston, MA 02115, USA (RID, ES, ECS, XM, AM)*

[4]*Medical School, University of Crete, Heraklion, Greece (ES).*

[5]*Southwest Research Institute, Boulder, CO 80302, USA (SC)*

[6]*Southwest Research Institute, San Antonio, TX 78238, USA (MAD, MID)*

[7]*University of San Antonio, San Antonio, TX 78249, USA (MAD, MID)*

[8]*Department of Radiation Oncology, University of California, Irvine, Orange, CA 92868, USA (CLL).*

Supplementary Table 1: Cumulative basal (4 mM K+) and high K+-evoked extracellular levels of DA, 5-HT, NE, Glu, and GABA in dialysate from PFC of mice exposed to acute or chronic simulated GCR versus controls. An unpaired two-tailed t-test was used to determine differences in cumulative increases in neurotransmitter levels among groups.

**K+ (mM)**	**Condition**	**DA nM (±S.E.M)**	**5-HT nM (±S.E.M)**	**NE nM (±S.E.M)**	**Glu nM (±S.E.M)**	**GABA nM (±S.E.M)**
**4**	Control	0.28 (± 0.06)	0.24 (± 0.04)	0.93 (± 0.10)	671.2 (± 85.2)	37.9 (± 13.5)
	Acute	0.76 (± 0.15)<br>*$t(78) = 2.88, P = 0.005$	0.42 (± 0.06)<br>*$t(78) = 2.33, P = 0.022$	0.68 (± 0.11)	635.4 (± 54.3)	74.6 (± 14.5)
	Chronic	0.60 (± 0.13)<br>*$t(70) = 2.43, P = 0.018$	0.47 (± 0.06)<br>*$t(70) = 3.01, P = 0.004$	1.49 (± 0.20)<br>*$t(70) = 2.59, P = 0.012$<br>#$t(70) = 3.72, P = 0.0004$	923.5 (± 141.6)<br>#$t(70) = 2.05, P = 0.044$	50.9 (± 12.1)
**30**	Control	0.91 (± 0.18)	0.43 (± 0.04)	2.12 (± 0.23)	1074.3 (± 113.4)	61.80 (± 17.17)
	Acute	1.71 (± 0.31)<br>*$t(78) = 2.24, P = 0.028$	0.80 (± 0.14)<br>*$t(78) = 2.59, P = 0.011$	2.04 (± 0.35)	1110.0 (± 97.2)	114.34 (± 20.15)
	Chronic	1.45 (± 0.28)	0.92 (± 0.14)<br>*$t(70) = 3.80, P = 0.0003$	2.97 (± 0.32)<br>*$t(70) = 2.20, P = 0.031$	1560.4 (± 237.2)	92.81 (± 18.10)
**60**	Control	1.51 (± 0.28)	0.68 (± 0.03)	3.62 (± 0.41)	1384.1 (± 141.4)	101.03 (± 22.35)
	Acute	2.83 (± 0.46)<br>*$t(78) = 2.44, P = 0.017$	1.21 (± 0.20)<br>*$t(78) = 2.58, P = 0.012$	3.38 (± 0.49)	1520.1 (± 135.3)	167.48 (± 26.47)
	Chronic	2.43 (± 0.43)	1.49 (± 0.21)<br>*$t(70) = 4.24, P < 0.0001$	4.90 (± 0.43)<br>*$t(70) = 2.16, P = 0.034$<br>#$t(70) = 2.27, P = 0.026$	2094.8 (± 291.1)<br>*$t(70) = 2.34, P = 0.022$	173.32 (± 28.02)<br>*$t(70) = 2.04, P = 0.045$
**120**	Control	2.21 (± 0.40)	1.22 (± 0.06)	5.32 (± 0.54)	1939.9 (± 186.7)	265.74 (± 45.16)
	Acute	4.26 (± 0.63)<br>*$t(78) = 2.75, P = 0.008$	1.73 (± 0.26)	5.26 (± 0.74)	2049.4 (± 173.8)	315.07 (± 48.91)
	Chronic	3.75 (± 0.62)<br>*$t(70) = 2.16, P = 0.034$	2.28 (± 0.31)<br>*$t(70) = 3.68, P = 0.0005$	7.15 (± 0.55)<br>*$t(70) = 2.34, P = 0.022$	3193.0 (± 414.3)<br>*$t(70) = 2.95, P = 0.004$<br>#$t(70) = 2.74, P = 0.008$	511.84 (± 85.51)<br>*$t(70) = 2.69, P = 0.009$<br>#$t(70) = 2.09, P = 0.039$

Data were calculated as the cumulative levels of the four consecutive samples from all subjects after stimulation with 4 mM K+. * $P < 0.05$ indicates signifcant difference beween acute or chronic GCR exposure groups vs controls; # $P < 0.05$ indicates significant difference between acute vs chronic GCR exposure.

Supplementary Table 2. Pearson correlations analysis, statistical values, and number of samples for neurotransmitters quantified in control and acute or chronic GCR exposed mice during 4 mM K+ stimulation.

Condition	Neurotransmitter 1	Neurotransmitter 2	Pearson correlation	95% CI	*P*-value	Samples (n)
Control	DA	5-HT	-0.196	-0.4785 to 0.1228	0.225	40
	DA	NE	-0.158	-0.4473 to 0.1617	0.331	40
	DA	Glu	-0.241	-0.5141 to 0.07595	0.134	40
	DA	GABA	-0.158	-0.4472 to 0.1618	0.331	40
	5-HT	NE	0.368	0.06346 to 0.6094	0.020	40
	5-HT	Glu	0.104	-0.2143 to 0.4026	0.522	40
	5-HT	GABA	0.063	-0.2531 to 0.3676	0.698	40
	NE	Glu	-0.013	-0.3229 to 0.3001	0.938	40
	NE	GABA	0.116	-0.2027 to 0.4127	0.475	40
	Glu	***GABA***	***0.908***	***0.8326 to 0.9509***	***5.651e-016***	***40***
Acute	DA	5-HT	0.307	-0.005090 to 0.5645	0.054	40
	DA	NE	-0.243	-0.5155 to 0.07406	0.131	40
	DA	Glu	-0.229	-0.5044 to 0.08905	0.156	40
	DA	GABA	-0.230	-0.5053 to 0.08781	0.153	40
	5-HT	NE	-0.049	-0.3552 to 0.2665	0.763	40
	5-HT	Glu	0.252	-0.06417 to 0.5228	0.116	40
	5-HT	GABA	0.125	-0.1941 to 0.4201	0.442	40
	NE	Glu	-0.101	-0.4003 to 0.2169	0.533	40
	NE	GABA	0.179	-0.1399 to 0.4650	0.268	40
	Glu	GABA	0.443	0.1524 to 0.6629	0.004	40
Chronic	DA	5-HT	-0.094	-0.4289 to 0.2631	0.608	32
	DA	***NE***	***0.692***	***0.4525 to 0.8384***	***0.00001***	***32***
	DA	Glu	-0.341	-0.6166 to 0.008440	0.056	32
	DA	GABA	-0.328	-0.6073 to 0.02333	0.067	32
	5-HT	NE	-0.448	-0.6889 to -0.1173	0.010	32
	5-HT	Glu	-0.109	-0.4405 to 0.2496	0.554	32
	5-HT	GABA	-0.226	-0.5330 to 0.1328	0.213	32
	NE	Glu	-0.291	-0.5805 to 0.06459	0.107	32
	NE	GABA	-0.154	-0.4769 to 0.2060	0.401	32
	Glu	***GABA***	***0.760***	***0.5595 to 0.8764***	***4.544e-007***	***32***

Supplementary Table 3. Pearson correlations analysis, statistical values, and number of samples for neurotransmitters quantified in control and acute or chronic GCR exposed mice during 30 mM K+ stimulation.

Condition	Neurotransmitter 1	Neurotransmitter 2	Pearson correlation	95% CI	*P*-value	Samples (n)
Control	DA	5-HT	0.034	-0.2805 to 0.3418	0.835	40
	DA	NE	0.002	-0.3098 to 0.3132	0.991	40
	DA	Glu	0.102	-0.2167 to 0.4005	0.532	40
	DA	GABA	0.238	-0.07984 to 0.5112	0.140	40
	5-HT	NE	0.294	-0.01968 to 0.5544	0.066	40
	5-HT	Glu	-0.101	-0.4001 to 0.2171	0.534	40
	5-HT	GABA	0.117	-0.2021 to 0.4132	0.473	40
	NE	Glu	-0.229	-0.5044 to 0.08902	0.156	40
	NE	GABA	0.043	-0.2725 to 0.3495	0.794	40
	Glu	GABA	0.227	-0.09085 to 0.5030	0.159	40
Acute	DA	5-HT	0.483	0.2018 to 0.6906	0.002	40
	DA	***NE***	***0.695***	***0.4901 to 0.8277***	***6.340e-007***	***40***
	DA	Glu	0.434	0.1411 to 0.6564	0.005	40
	DA	GABA	0.273	-0.04158 to 0.5391	0.087	40
	5-HT	NE	0.469	0.1846 to 0.6811	0.002	40
	5-HT	Glu	0.445	0.1555 to 0.6647	0.004	40
	5-HT	GABA	0.302	-0.01067 to 0.5606	0.058	40
	NE	***Glu***	***0.534***	***0.2669 to 0.7249***	***3.868e-004***	***40***
	NE	GABA	0.218	-0.09990 to 0.4962	0.176	40
	Glu	***GABA***	***0.533***	***0.2653 to 0.7241***	***4.012e-004***	***40***
Chronic	***DA***	***5-HT***	***0.582***	***0.2922 to 0.7735***	***0.0005***	***32***
	DA	***NE***	***0.543***	***0.2397 to 0.7497***	***0.001***	***32***
	DA	Glu	-0.229	-0.5353 to 0.1296	0.206	32
	DA	GABA	0.381	0.03696 to 0.6439	0.032	32
	5-HT	NE	0.396	0.05505 to 0.6544	0.025	32
	5-HT	Glu	-0.005	-0.3533 to 0.3441	0.977	32
	5-HT	GABA	0.269	-0.08746 to 0.5651	0.136	32
	NE	Glu	-0.386	-0.6476 to -0.04317	0.029	32
	NE	GABA	0.146	-0.2133 to 0.4709	0.424	32
	Glu	***GABA***	***0.517***	***0.2059 to 0.7337***	***0.002***	***32***

Supplementary Table 4. Pearson correlations analysis, statistical values, and number of samples for neurotransmitters quantified in control and acute or chronic GCR exposed mice during 60 mM K+ stimulation.

Condition	Neurotransmitter 1	Neurotransmitter 2	Pearson correlation	95% CI	*P*-value	Samples (n)
Control	DA	5-HT	0.303	-0.009186 to 0.5617	0.057	40
	DA	NE	0.275	-0.04032 to 0.5400	0.086	40
	DA	Glu	-0.100	-0.3993 to 0.2180	0.538	40
	DA	GABA	0.183	-0.1363 to 0.4678	0.259	40
	5-HT	NE	0.453	0.1643 to 0.6697	0.003	40
	5-HT	Glu	-0.030	-0.3382 to 0.2844	0.855	40
	5-HT	GABA	0.114	-0.2047 to 0.4109	0.484	40
	NE	Glu	0.011	-0.3014 to 0.3215	0.946	40
	NE	GABA	0.084	-0.2336 to 0.3854	0.606	40
	Glu	GABA	0.475	0.1915 to 0.6849	0.002	40
Acute	DA	5-HT	0.467	0.1820 to 0.6796	0.002	40
	DA	***NE***	***0.567***	***0.3106 to 0.7468***	***0.0001***	***40***
	DA	Glu	-0.250	-0.5209 to 0.06672	0.120	40
	DA	GABA	0.210	-0.1085 to 0.4896	0.193	40
	5-HT	NE	0.492	0.2136 to 0.6970	0.0012	40
	5-HT	Glu	-0.097	-0.3965 to 0.2213	0.552	40
	5-HT	GABA	0.488	0.2082 to 0.6940	0.001	40
	NE	Glu	0.028	-0.2859 to 0.3367	0.863	40
	NE	***GABA***	***0.599***	***0.3541 to 0.7676***	***4.362e-005***	***40***
	Glu	GABA	0.113	-0.2061 to 0.4098	0.489	40
Chronic	***DA***	***5-HT***	***0.677***	***0.4292 to 0.8296***	***0.00002***	***32***
	DA	NE	0.111	-0.2471 to 0.4427	0.545	32
	DA	Glu	-0.305	-0.5906 to 0.04923	0.090	32
	DA	GABA	-0.039	-0.3827 to 0.3137	0.831	32
	5-HT	NE	0.185	-0.1753 to 0.5011	0.312	32
	5-HT	Glu	-0.237	-0.5411 to 0.1217	0.191	32
	5-HT	GABA	0.049	-0.3047 to 0.3912	0.789	32
	NE	Glu	-0.215	-0.5245 to 0.1443	0.237	32
	NE	GABA	0.059	-0.2956 to 0.3996	0.748	32
	Glu	***GABA***	***0.542***	***0.2389 to 0.7494***	***0.001***	***32***

Supplementary Table 5. Pearson correlations analysis, statistical values, and number of samples for neurotransmitters quantified in control and acute or chronic GCR exposed mice. Individual values during 120 mM K+ stimulation.

Condition	Neurotransmitter 1	Neurotransmitter 2	Pearson correlation	95% CI	*P*-value	Samples (n)
Control	DA	5-HT	-0.214	-0.4930 to 0.1040	0.184	40
	DA	NE	0.093	-0.2246 to 0.3935	0.566	40
	DA	Glu	-0.241	-0.5136 to 0.07669	0.135	40
	DA	GABA	0.004	-0.3082 to 0.3148	0.982	40
	5-HT	NE	0.141	-0.1784 to 0.4334	0.386	40
	5-HT	Glu	0.400	0.1015 to 0.6329	0.010	40
	5-HT	GABA	0.261	-0.05464 to 0.5297	0.103	40
	NE	Glu	0.234	-0.08373 to 0.5084	0.146	40
	NE	GABA	0.031	-0.2832 to 0.3392	0.849	40
	Glu	***GABA***	***0.560***	***0.3013 to 0.7422***	***1.704e-004***	***40***
Acute	***DA***	***5-HT***	***0.598***	***0.3564 to 0.7652***	***0.00004***	***40***
	DA	***NE***	***0.782***	***0.6245 to 0.8783***	***1.581e-009***	***40***
	DA	Glu	0.064	-0.2486 to 0.3644	0.691	40
	DA	***GABA***	***0.664***	***0.4478 to 0.8068***	***0.000002***	***40***
	5-HT	NE	0.387	0.09001 to 0.6207	0.012	40
	5-HT	Glu	0.094	-0.2196 to 0.3908	0.557	40
	5-HT	***GABA***	***0.586***	***0.3401 to 0.7574***	***0.000056***	***40***
	NE	Glu	0.350	0.04697 to 0.5934	0.025	40
	NE	GABA	0.710	0.5155 to 0.8355	1.957e-007	40
	Glu	GABA	0.394	0.09876 to 0.6261	0.011	40
Chronic	***DA***	***5-HT***	***0.647***	***0.3851 to 0.8124***	***0.00006***	***32***
	DA	NE	0.182	-0.1781 to 0.4989	0.319	32
	DA	Glu	-0.076	-0.4135 to 0.2803	0.680	32
	DA	GABA	0.054	-0.3000 to 0.3956	0.767	32
	5-HT	NE	0.167	-0.1933 to 0.4871	0.362	32
	5-HT	Glu	0.104	-0.2535 to 0.4372	0.569	32
	5-HT	***GABA***	***0.510***	***0.1959 to 0.7289***	***0.003***	***32***
	NE	Glu	0.016	-0.3345 to 0.3627	0.930	32
	NE	GABA	0.179	-0.1808 to 0.4968	0.326	32
	Glu	***GABA***	***0.579***	***0.2885 to 0.7718***	***0.001***	***32***
