# Supplementary material for: Complex 33-beam simulated galactic cosmic radiation exposure impacts cognitive function and prefrontal cortex neurotransmitter networks in male mice
Source: Nat Commun. 2023 Nov 27;14:7779. doi: 10.1038/s41467-023-42173-x (PMC10682413; doi:10.1038/s41467-023-42173-x)
Supplement: Supplementary file 3 — Reporting Summary [file 41467_2023_42173_MOESM3_ESM.pdf]

nature portfolio

Corresponding author(s): Rajeev I. Desai, Ph.D
Department of Psychiatry, Harvard Medical School
Behavioral Biology Program, McLean Hospital
115 Mill Street
Belmont, MA 02478, USA
E-mail: rdesai@mclean.harvard.edu
Telephone: (617) 855-3303

Last updated by author(s): Aug 11, 2023

Reporting Summary

Nature Portfolio wishes to improve the reproducibility of the work that we publish. This form provides structure for consistency and transparency in reporting. For further information on Nature Portfolio policies, see our Editorial Policies and the Editorial Policy Checklist.

Statistics

For all statistical analyses, confirm that the following items are present in the figure legend, table legend, main text, or Methods section.

n/a	Confirmed	
☐	☒	The exact sample size (*n*) for each experimental group/condition, given as a discrete number and unit of measurement
☐	☒	A statement on whether measurements were taken from distinct samples or whether the same sample was measured repeatedly
☐	☒	The statistical test(s) used AND whether they are one- or two-sided<br>*Only common tests should be described solely by name; describe more complex techniques in the Methods section.*
☐	☒	A description of all covariates tested
☐	☒	A description of any assumptions or corrections, such as tests of normality and adjustment for multiple comparisons
☐	☒	A full description of the statistical parameters including central tendency (e.g. means) or other basic estimates (e.g. regression coefficient) AND variation (e.g. standard deviation) or associated estimates of uncertainty (e.g. confidence intervals)
☐	☒	For null hypothesis testing, the test statistic (e.g. *F*, *t*, *r*) with confidence intervals, effect sizes, degrees of freedom and *P* value noted<br>*Give P values as exact values whenever suitable.*
☒	☐	For Bayesian analysis, information on the choice of priors and Markov chain Monte Carlo settings
☒	☐	For hierarchical and complex designs, identification of the appropriate level for tests and full reporting of outcomes
☐	☒	Estimates of effect sizes (e.g. Cohen's *d*, Pearson's *r*), indicating how they were calculated

Our web collection on statistics for biologists contains articles on many of the points above.

Software and code

Policy information about availability of computer code

Data collection	All neurocognitive experimental events and data collection were programmed in E-Prime Professional 2.0 (Psychology Software Tools, Inc., Sharpsburg, PA). All microdialysis samples were analyzed on Thermo Fisher TSQ Altis Triple Quadrupole mass spectrometer with a Thermo Fisher Vanquish UHPLC at the front end. The derivatized neurotransmitters were first separated on Phenomenex C18 Kinetex F5 column 912 (150 X 4.6mm, 2.6 µm) and then analyzed by mass spectrometer operated in ESI-positive multiple reaction monitoring mode. Thermo TraceFinder Forensic LC was used to analyze neurotransmitter peaks. All statistical analysis were performed using GraphPad Prism, version 9.1.3. All of this information has been included in the manuscript.
Data analysis	We used open-source Python packages such as Numpy (1.20.3), Matplotlib (3.5.0), pandas (1.4.1), iRF (https://github.com/Yu-Group/iterative-Random-Forest) and Networkx (2.6.3) to analyze and visualize the data. Our codes are available through GitHub: https://github.com/subhamoysgit/neurotransmitter_networks. Correspondence and requests for materials should be addressed to Dr. Rajeev I. Desai.

For manuscripts utilizing custom algorithms or software that are central to the research but not yet described in published literature, software must be made available to editors and reviewers. We strongly encourage code deposition in a community repository (e.g. GitHub). See the Nature Portfolio guidelines for submitting code & software for further information.

Data

Policy information about availability of data

All manuscripts must include a data availability statement. This statement should provide the following information, where applicable:

- Accession codes, unique identifiers, or web links for publicly available datasets
- A description of any restrictions on data availability
- For clinical datasets or third party data, please ensure that the statement adheres to our policy

All data in the manuscript or the supplementary material are available from the corresponding author upon request. Correspondence and requests for materials should be addressed to Rajeev I. Desai.

Human research participants

Policy information about studies involving human research participants and Sex and Gender in Research.

Reporting on sex and gender	Not Applicable
Population characteristics	Not Applicable
Recruitment	Not Applicable
Ethics oversight	Not Applicable

Note that full information on the approval of the study protocol must also be provided in the manuscript.

Field-specific reporting

Please select the one below that is the best fit for your research. If you are not sure, read the appropriate sections before making your selection.

☒ Life sciences ☐ Behavioural & social sciences ☐ Ecological, evolutionary & environmental sciences

For a reference copy of the document with all sections, see nature.com/documents/nr-reporting-summary-flat.pdf

Life sciences study design

All studies must disclose on these points even when the disclosure is negative.

Sample size	Sample sizes were not precalculated. Due to the limited access to the complex 33 beam GCR simulation, animal numbers were fixed beforehand at 8-10/cohort. Historical data has indicated that radiation-induced group differences can be substantial and that a sample size of 8/group provides a power level of 80% for our behavioral and in vivo microdialysis studies.
Data exclusions	No data were excluded from the analysis
Replication	Replication of the given cohorts was not possible due to the limited access of the 33 beam GCR simulation. Longitudinal behavioral testing and in vivo microdialysis did yield robust data with minimal sample to sample variance. Behavioral testing and microdialysis studies were conducted independently. Behavioral testing was conducted first over a four month period in these subjects and it was not possible to replicate these studies due to the valuable nature of these GCR exposed subjects as well as the study goals which required the need to conduct within-subject neurotransmitter analysis using in vivo microdialysis techniques immediately after behavioral testing. Data for in vivo microdialysis experiments were not replicated as after sample collection, all subjects were sacrificed and histology was performed to verify probe placement in the PFC.
Randomization	All animals were randomly assigned to experimental groups, no significant differences between the weights of experimental subjects were noted that would skew random group allocations.
Blinding	Investigators were blinded to cohort allocations during data acquisition and subsequent analysis.

Reporting for specific materials, systems and methods

We require information from authors about some types of materials, experimental systems and methods used in many studies. Here, indicate whether each material, system or method listed is relevant to your study. If you are not sure if a list item applies to your research, read the appropriate section before selecting a response.

Materials & experimental systems

n/a	Involved in the study
☒	☐ Antibodies
☒	☐ Eukaryotic cell lines
☒	☐ Palaeontology and archaeology
☐	☒ Animals and other organisms
☒	☐ Clinical data
☒	☐ Dual use research of concern

Methods

n/a	Involved in the study
☒	☐ ChIP-seq
☒	☐ Flow cytometry
☒	☐ MRI-based neuroimaging

Animals and other research organisms

Policy information about studies involving animals; ARRIVE guidelines recommended for reporting animal research, and Sex and Gender in Research

Laboratory animals	Male C57BL/6 mice ?6-9 months old
Wild animals	The study did not involve wild animals.
Reporting on sex	All experiments were conducted in Male C57BL/6 mice. Due to limitations in exposure to simulated space radiation at Brookhaven National Laboratory the present studies were only conducted in male subjects.
Field-collected samples	The study did not involve samples collected from the field.
Ethics oversight	All animal procedures were approved by the Institutional Animal Care and Use Committee at McLean Hospital and NASA Space Radiation Laboratory (NSRL) at Brookhaven National Laboratory and were conducted in accordance with guidelines provided by the Committee on Care and Use of Laboratory Animals of the Institute of Laboratory Animal Resources, Commission on Life Sciences, National Research Council. Mice were housed in a temperature- (~18-23°C) and humidity (40-60%)-controlled vivarium with a 12-hour light/dark cycle (lights on from 7 AM to 7 PM). This text has been included in the manuscript.

Note that full information on the approval of the study protocol must also be provided in the manuscript.
